# The ViaHole technique: a novel approach for recanalizing major side branch occluded by Viabahn stent-graft

**DOI:** 10.1186/s42155-023-00385-8

**Published:** 2023-07-15

**Authors:** Takuya Haraguchi, Masanaga Tsujimoto, Ryo Otake, Yoshifumi Kashima, Katsuhiko Sato, Tsutomu Fujita

**Affiliations:** Department of Cardiology, Asia Medical Group, Sapporo Heart Center, Sapporo Cardio Vascular Clinic, North 49, East 16, 8-1, Higashi Ward, Sapporo, Hokkaido 007-0849 Japan

**Keywords:** Stent-graft, Viabahn, Recanalization, Complication management, Endovascular treatment, Peripheral artery disease, Peripheral intervention

## Abstract

**Introduction:**

In managing arterial rupture, stent-graft implantation may cause limb ischemia by crossing a major branch for hemostasis. The ViaHole technique could circumvent a major branch occlusion.

**Materials and methods:**

The process involved advancing retrograde devices into an occluded major branch by the stent-graft implantation to reach the outer surface of the stent-graft, puncturing the stent-graft with a 20-gauge needle to touch the retrograde device, manipulating the guidewire through the needle hole and externalizing it, advancing the microcatheter into the proximal lumen, catching the microcatheter using an antegrade 4-Fr catheter, inserting an antegrade guidewire into the retrograde microcatheter to cross the stent-graft hole, dilating the lesion and stent-graft hole using a 3.0-mm balloon, and ensuring hemostasis at the puncture site.

**Results:**

A 72-year-old male with a history of stent-grafted treatment for right popliteal aneurysm presented with acute limb ischemia (ALI). The occlusion spanned distal superficial femoral artery to the below-the-knee arteries. Hemostasis was achieved after an unintentional rupture of the proximal posterior tibial artery during surgical thrombectomy by implanting endoluminal stent-grafts instead of surgical bypass due to no distal anastomosis site. However, recurrent ALI occurred three months later. Surgical bypass was again deemed unfeasible due to no run-off. Unsuccessful recanalization attempts of the bilateral tibial arteries led us to perform the ViaHole technique to recanalize the peroneal artery occlusion. Finally. successful revascularization was achieved, and 1-year patency was confirmed.

**Conclusions:**

The ViaHole technique may be valuable for revascularizing a major side branch occluded by stent-graft implantation.

**Supplementary Information:**

The online version contains supplementary material available at 10.1186/s42155-023-00385-8.

## Introduction

Heparin-bonded endoluminal stent-graft (Viabahn: W.L. Gore & Associates, AZ) with expanded polytetrafluoroethylene (ePTFE) covering has been reported as valuable in managing arterial rupture [1]. However, in some cases, stent-graft implantation may necessitate crossing a significant side branch to achieve hemostasis, potentially resulting in major branch occlusion and subsequent limb ischemia [2]. We introduce a novel recanalization method termed the ViaHole technique to address this issue.

## Main text

### Materials and methods

Firstly, retrograde devices are advanced into a major branch occluded by stent-graft implantation. The retrograde devices reach the outer surface of the stent-graft, and a 20-gauge needle is used to puncture the stent-graft and access the retrograde device. The next step is to advance the guidewire through the needle hole to out of the body. After externalizing the guidewire, the retrograde microcatheter is advanced into the proximal lumen. An antegrade 4-Fr catheter is used to catch the retrograde microcatheter, and an antegrade guidewire is then inserted into the retrograde microcatheter to cross the stent-graft hole. The lesion and stent-graft hole are dilated using a balloon. Finally, it is crucial to ensure hemostasis at the puncture site.

**Fig. 1:**
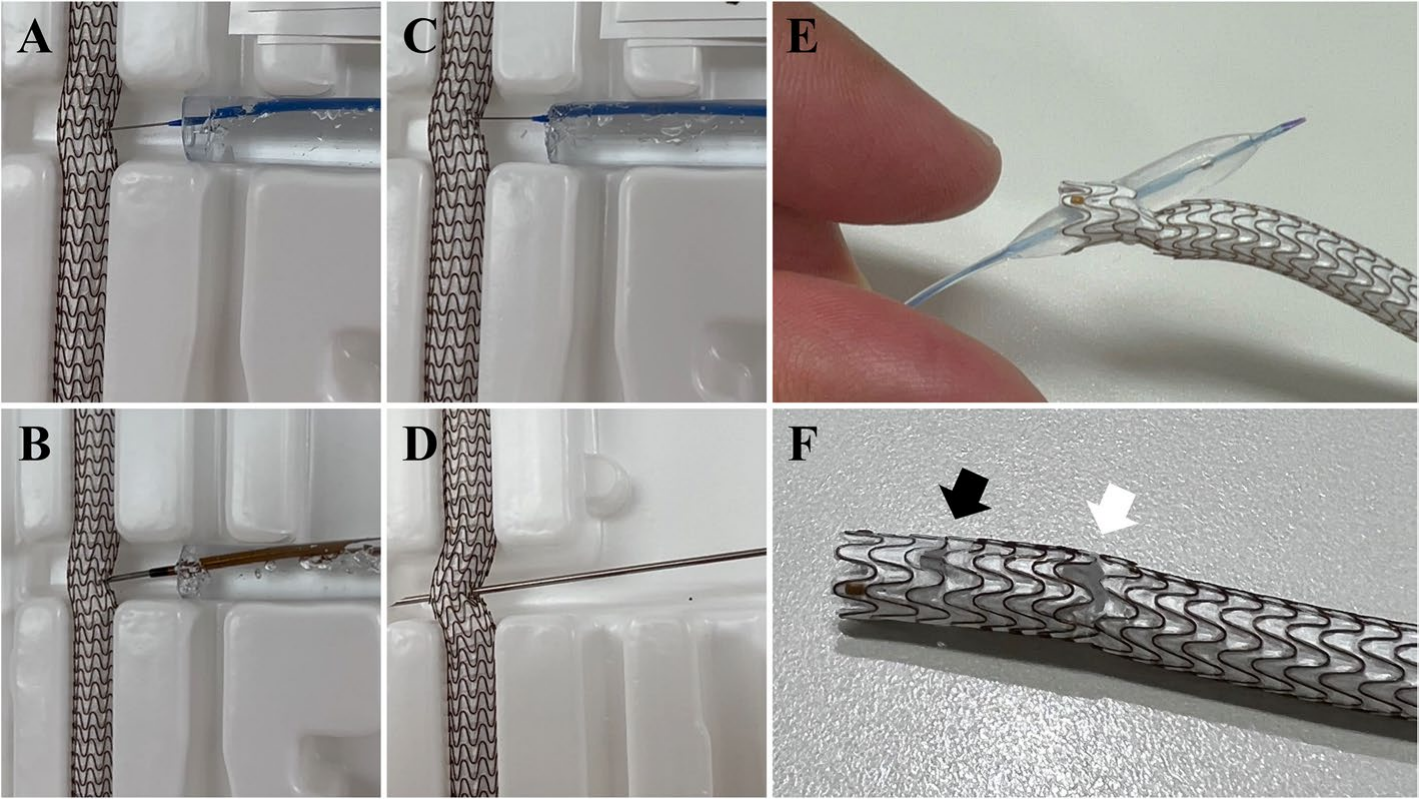
In vitro experiments assessing the performance of various devices. The performance of devices in penetrating the ePTFE of Viabahn stent-graft and the state of holes in the ePTFE created by balloon expansion were assessed. **A**–**C** Unsuccessful penetration into stent-graft using the hardest 0.014-inch tapered type guidewire, guidewire tail, and Wingman (Reflow Medical Inc., San Clemente, USA), with a needle at the tip of the catheter; and **D** successful penetration into the stent-graft using a 20-gauge needle; and **E**, **F** holes created by 3.0 mm (black arrow) and 4.0 mm balloons (white arrow) in the ePTFE site on the stent-graft remaining open without acute recoil

To verify the reproducibility of the ViaHole technique, in vitro experiments were performed using the same 5.0 × 100-mm Viabahn used in this case to determine whether its ePTFE could be punctured with puncture devices, whether all parts of the ePTFE could be punctured, and whether the ePTFE could be opened after balloon dilatation of the puncture hole. As a result, a 20-gauge needle successfully penetrated all parts of the ePTFE, including the edges, whereas the hardest 0.014-inch tapered type guidewire, guidewire tail, and Wingman (Reflow Medical Inc., USA), with a needle at the tip of the catheter, did not penetrate it (Fig. 1A–D) (Supplemental movie 1). Furthermore, holes of the ePTFE, created by 3.0- and 4.0-mm balloons, remained open without acute occlusion (Fig. 1E, F). These vitro experimental results indicated that this technique might rescue a major branch occluded by stent-graft implantation.

## Results

**Fig. 2:**
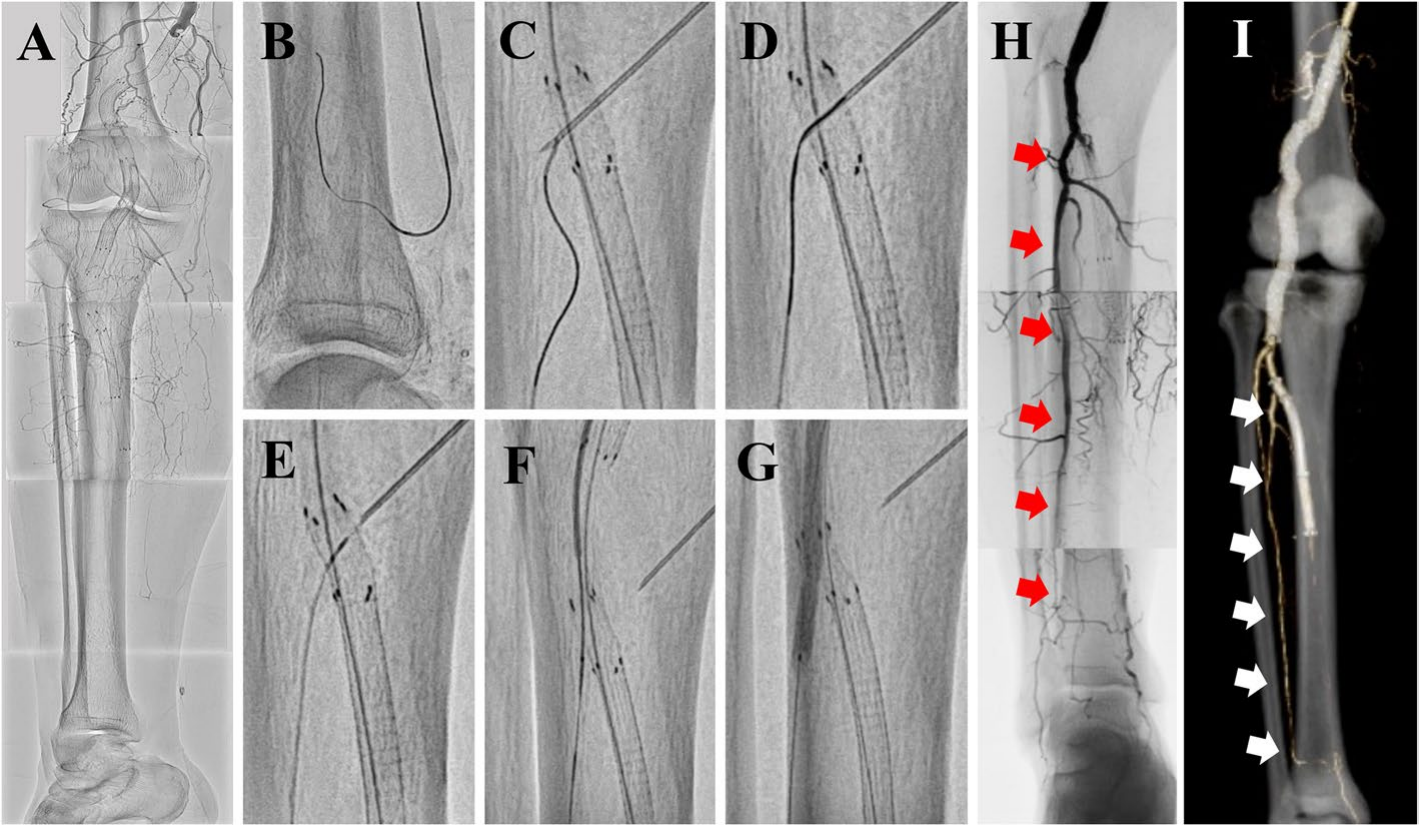
Overview and results of the ViaHole technique. **A** Initial angiogram showing thrombotic occlusion due to acute limb ischemia; **B** advancement of retrograde guidewire and microcatheter into the peroneal artery occlusion from distal posterior tibial artery via the posterior perforating branch of the peroneal artery; **C** successful puncture of the stent-graft with a 20-gauge needle, touching the tip of the retrograde microcatheter; **D** externalization of the retrograde guidewire through needle hole; **E** forceful advancement of the retrograde microcatheter over the externalized guidewire across the posterior wall of the stent-graft into the tibioperoneal trunk; **F** insertion of the antegrade guidewire into the retrograde microcatheter and advancement of the antegrade microcatheter into the peroneal artery; **G** dilation of the lesion, including the expanded polytetrafluoroethylene hole, using a 3.0 mm noncompliant balloon; **H** angiogram revealing successful creation of the stent-graft hole and sufficient flow from the peroneal artery (red arrows) to the below-the-ankle arteries; and **I** 1-year computed tomography angiogram demonstrating patency from the superficial femoral artery to the peroneal artery (white arrows)

A 72-year-old male, with a history of right popliteal aneurysm treatment involving stent-graft implantation, presented with sudden pain and paresthesia in his right foot due to acute limb ischemia (ALI). Computed tomography angiogram (CTA) and angiography revealed occlusion from the distal superficial femoral artery (SFA) to the below-the-knee arteries. During attempts to remove the thrombus using surgical thrombectomy, an unintentional rupture occurred in the proximal posterior tibial artery (PTA). A surgical bypass was not employed due to no run-off in the below-the-knee arteries. Therefore, an endoluminal stent-graft was emergently inserted to achieve hemostasis in the proximal PTA without covering the peroneal artery; however, bleeding persisted. Consequently, another 5.0 × 100-mm stent-graft was placed up to the tibioperoneal trunk, resulting in the occlusion of the peroneal artery. To prevent ALI reoccurrence, anticoagulation (Warfarin) and dual antiplatelet drugs were prescribed. Nevertheless, the patient experienced recurrent ALI three months later (Fig. 2A). A surgical bypass was again deemed unfeasible due to no run-off in below-the-knee arteries by initial angiography. After unsuccessful recanalization attempts for both tibial arteries involving surgical thrombectomy and 3.0-mm balloon dilatation, the decision was made to recanalize the peroneal artery occlusion using the ViaHole technique for limb salvage.

First, a retrograde guidewire and microcatheter were advanced into the peroneal artery occlusion from the distal PTA through the posterior perforating branch of the peroneal artery (Fig. 2B). Once the retrograde devices reached the peroneal arterial ostium and touched the outer surface of the posterior wall of ePTFE on the stent-graft. To access the retrograde guidewire or microcatheter in the peroneal orifice, a 20-gauge needle was used to puncture the stent-graft, passing through the anterior wall of ePTFE, the tibioperoneal trunk with confirming blood return, and the posterior wall of ePTFE (Fig. 2C). Subsequently, the retrograde guidewire was manipulated and pulled out through the hole to externalize the guidewire (Fig. 2D). The retrograde microcatheter was advanced over the guidewire and through the hole in the ePTFE, reaching the tibioperoneal trunk (Fig. 2E). The retrograde microcatheter was then captured with a 4-Fr antegrade catheter, and an antegrade guidewire was inserted into the retrograde microcatheter to advance an antegrade microcatheter into the peroneal artery (Fig. 2F). To dilate the hole at the posterior wall of ePTFE and ensure hemostasis at the anterior wall of ePTFE, a 3.0-mm balloon was inflated (Fig. 2G). The final angiogram demonstrated sufficient blood flow through the peroneal artery to below-the-ankle arteries (Fig. 2H). The process of this technique is presented in the supplementary material (Supplemental movie 2). After the procedure, warfarin was changed to a direct oral anticoagulant (rivaroxaban 2.5 mg twice daily) to stabilize the effect of the prevention of thrombus development. Ultrasound and CTA confirmed patency at 1-, 6-, and 12-month after treatment (Fig. 2I).

## Discussion

Complex peripheral interventions have been associated with an increased risk of vascular complications. When addressing perforation near vessel bifurcation, stent-graft implantation across a major side branch may be unavoidable to achieve hemostasis. The neocarina reconstruction technique, involving a Brockenbrough needle to perforate the ePTFE of the stent-graft and 5.5-mm balloon dilatation, has been reported for reaccessing major branch [3]. However, the use of Brockenbrough needles for peripheral procedures is not covered by insurance, and their manipulation and procedural success depend on the anatomic course, the diameter of the vessel, and the hardness of the plaque. Although another method is to use a snare to grasp a re-entry wire to externalize a guidewire, in this case, a snare could not be opened because no lumen was in the peroneal artery occlusion, and the snare was difficult to bring in due to the trans-collateral approach. Contrasting this intravascular approach, the ViaHole technique is an extracorporeal approach that penetrates ePTFE with a 20-gauge needle, allowing for fine manipulation. This case demonstrated successful limb salvage utilizing the ViaHole technique. When scaffolds designed for small vessels are available, the hole could be reinforced, leading to long-term patency improvement. Therefore, dedicated devices are necessary to standardize the technique.

## Limitations

The ViaHole technique has several limitations: 1) the technique is applicable to infrainguinal arteries but not suprainguinal arteries; 2) this method is infeasible without a retrograde guidewire and microcatheter in the target lumen to be punctured with a needle; 3) while the current report showed revascularization of the peroneal artery, this technique should be used to specifically recanalize the profunda artery in cases where a stent-graft is placed from the common femoral artery to the SFA; and 4) the long-term clinical impact of this technique on restenosis remains uncertain.

## Conclusions

The ViaHole technique may be valuable for revascularizing a major side branch occluded by Viabahn stent-graft implantation for vessel perforation. Additional research is warranted to assess the success and long-term outcomes of this technique in various clinical scenarios.

## Supplementary Information

**Additional file 1.** In vitro experiments assessing the performance of various devices in penetrating the ePTFE of Viabahn stent-graft

**Additional file 2.** The process of the ViaHole technique

## Data Availability

The data are available from the corresponding author upon reasonable request.

## Abbreviations

ALI: Acute limb ischemia
CTA: Computed tomography angiogram
ePTFE: Expanded polytetrafluoroethylene
PTA: Posterior tibial artery
SFA: Superficial femoral artery
